# Light-Induced Surface Potential Modification in MoS_2_ Monolayers on Au Nanostripe Arrays

**DOI:** 10.1038/s41598-019-50950-2

**Published:** 2019-10-08

**Authors:** Soyeong Kwon, Min Hee Kwon, Jungeun Song, Eunah Kim, Youngji Kim, Bo Ra Kim, Jerome K. Hyun, Sang Wook Lee, Dong-Wook Kim

**Affiliations:** 1 Department of Physics, Ewha Womans University, Seoul, 03760 Korea; 2 Department of Chemistry and Nano Science, Ewha Womans University, Seoul, 03760 Korea

**Keywords:** Nanoscale materials, Electronic devices, Photonic devices

## Abstract

In this work, the surface potential (*V*_S_) of exfoliated MoS_2_ monolayers on Au nanostripe arrays with period of 500 nm was investigated using Kelvin probe force microscopy. The surface morphology showed that the suspended MoS_2_ region between neighboring Au stripes underwent tensile-strain. In the dark, the *V*_S_ of the MoS_2_ region on the Au stripe (*V*_S_-Au) was larger than that of the suspended MoS_2_ region (*V*_S_-S). However, under green light illumination, *V*_S_-Au became smaller than *V*_S_-S. To explain the *V*_S_ modification, band diagrams have been constructed taking into consideration not only the local strain but also the electronic interaction at the MoS_2_/Au interface. The results of this work provide a basis for understanding the electrical properties of MoS_2_-metal contacts and improving the performance of MoS_2_-based optoelectronic devices.

## Introduction

Atomic-layered transition metal dichalcogenide (TMD) materials have attracted growing attention from a fundamental scientific perspective and for practical applications. This is primarily because of their fascinating physical characteristics that originate from their unique two-dimensional nature^1^. Most TMD-based device architectures have metal thin films and nanostructures and they are applied as electrodes, catalysts, and plasmonic nanoantennas^1–10^. In particular, TMD-metal nanostructures have exhibited promising physical phenomena, including increased light emission intensity, emission of single photons, and control of valley-polarized excitons^5–10^.

Understanding the physical properties of the TMD/metal interface is crucial for reliable device operation and electrical characterizations^3^. The electronic interaction at the metal/TMD interface originates from the overlap of the electron wavefunctions of the two materials, which can modify the charge distribution at the interface. These phenomena have been intensively investigated for doping and contact resistance control of TMD materials^2,3^. Metal nanostructures on or under TMD layers also can cause elastic deformations in TMD materials, resulting in modification of their physical properties^7–9^. Bandgap reduction in strained TMD layers can confine photo-generated excitons to local regions, which leads to spatial control of radiative recombination and the achievement of quantum light emitters^9^. Experimental characterizations of TMD-metal nanostructures have been mainly carried out using optical spectroscopy techniques^5–10^. The optical excitation and response studies, however, cannot directly reveal the behavior of charge carriers in the nanostructures. It is highly desirable to provide direct information regarding the generation, recombination, and transport of charge carriers in TMD-based nanostructures.

Kelvin probe force microscopy (KPFM), a scanning-probe-based technique, has been widely used to measure local surface potential (*V*_S_)^11–21^. The *V*_S_ measurements of TMD materials are very useful to identify the number of layers^11–13^, study the photo-carrier generation processes^13,14^ and estimate the built-in potential at the heterojunctions^15,16^. Since surface adsorbed molecules, such as H_2_O and O_2_, can transfer charges to the TMD materials, the measurement environment and the sample preparation procedures can significantly vary the measured *V*_S_ of TMD materiaels^11–14,17,18^. The light exposure can cause not only the photo-carrier generation/separation but also charge transfer from/to adsorbed/desorbed molecules. All these processes should be considered to explain the light-induced *V*_S_ change^13,14,19,20^. Careful measurements and analyses of the *V*_S_ of TMD-metal nanostructures will enable us to achieve better understanding of their physical characteristics.

In this work, MoS_2_-Au nanostructures, which comprised MoS_2_ monolayers on periodic Au nanostripe arrays, were fabricated. The spatial distribution of *V*_S_ in the dark and under illumination was investigated via KPFM in dry N_2_ ambient. The periodic modulation of the surface topography suggested that the MoS_2_ monolayers underwent tensile strain because of the nanostripes. Band diagrams of the MoS_2_-Au nanostructures were proposed to explain the spatial distribution of the *V*_S_ and the influence of light. This work can aid in understanding how local strain and electronic interactions could affect the physical characteristics of MoS_2_-metal nanostructures.

## Results and Discussion

**Figure 1:**
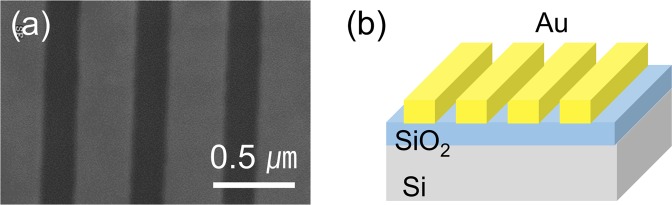
(**a**) Top view scanning electron micrograph and (**b**) a schematic diagram of a patterned Au stripe array on a SiO_2_/Si substrate.

Figure 1a,b show a top view scanning electron microscopy image and a schematic diagram of an Au stripe array, respectively. The Au stripes, consisting of 5-nm-thick Cr adhesion layers and 20-nm-thick Au layers, were fabricated on SiO_2_/Si substrates using electron-beam lithography patterning and electron-beam evaporation, followed by lift-off processes. The period and total area of the Au stripe arrays were 0.5 μm and 15 × 15 μm^2^, respectively. MoS_2_ flakes were mechanically exfoliated from the bulk on 300-nm-SiO_2_/Si substrates, and then transferred to the Au stripe arrays using a typical wet-transfer technique^14^. The charge transfer and Fermi level shifts at the metal/MoS_2_ contacts have been investigated using MoS_2_-based devices with metal nanoparticles^2,18^, and electrodes^3,13,14^. The MoS_2_ surface should have inhomogeneity, due to contaminants^17^, local ripples^21^, and structural/chemical imperfections. Thus, regularly shaped and spaced metal stripes will help us to clearly distinguish the periodic features originated from metal/MoS_2_ contacts from randomly distributed extrinsic ones.

**Figure 2:**
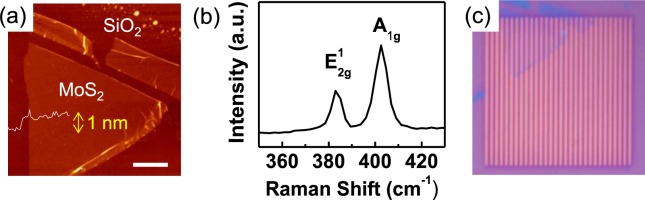
(**a**) An AFM topography image (area: 10 × 10 μm^2^) with 2 μm scale bar and a height profile and (**b**) a Raman spectrum of a MoS_2_ monolayer on a SiO_2_/Si substrate. (**c**) An optical microscopy image of the MoS_2_ monolayer transferred on an Au stripe array (area: 15 × 15 μm^2^).

Figure 2a shows the topography and height profile of an exfoliated MoS_2_ monolayer on an SiO_2_/Si substrate obtained via atomic force microscopy (AFM) measurements. An AFM system (Park Systems, XE-100) was used to obtain the surface topography of the sample. Room-temperature micro-Raman spectra were obtained using a 532 nm diode laser focused on a ~1 μm diameter spot. The Raman spectrum of a MoS_2_ flake shows the in-plane (${{\rm{E}}}_{2{\rm{g}}}^{1}$) and out-of-plane (A_1g_) vibration mode peaks at 383.2 and 402.7 cm^−1^, respectively (Fig. 2b). The thickness (~1 nm) and the separation between the two Raman peaks (19.5 cm^−1^) confirm the preparation of a monolayer MoS_2_ flake^1,14^. Figure 2c shows optical microscopy images of the MoS_2_ monolayer transferred to an Au stripe array. The shape of the flake was identical to that in the AFM image (Fig. 2a), indicating successful transfer of the specific flake to the target substrate.

**Figure 3:**
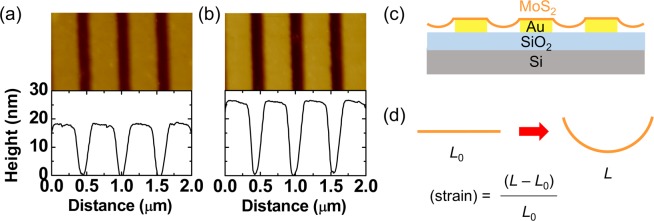
(**a**) AFM topography images and height profiles of (**a**) a MoS_2_ monolayer on an Au stripe array and (**b**) a bare Au stripe array. (**c**) A schematic cross-sectional view of a MoS_2_ monolayer on an Au stripe array. (**d**) A schematic illustration of the method to estimate strain in the samples. *L*_0_ and *L* indicate the original and strained MoS_2_ length between the neighboring Au stripes, respectively.

Figure 3a,b show AFM topography images and height profiles of a MoS_2_-monolayer-transferred Au stripe array and a bare one, respectively. The MoS_2_ monolayer on the Au stripe array shows periodic topographic modulation, just like the bare Au stripe array. The peak-to-valley height of the MoS_2_-transferred Au stripe array was smaller than that of the bare Au stripe. This suggests that the exfoliated MoS_2_ monolayer adheres to the edge of the Au stripe and the MoS_2_ region between the neighboring stripes is suspended. Similar results have been reported in MoS_2_ layers transferred on pre-patterned substrates^22,23^.

A cross-sectional view of the sample is schematically illustrated in Fig. 3c and the MoS_2_ monolayer in the suspended region undergoes uniaxial tensile strain. The original and strained MoS_2_ length is *L*_0_ and *L*, respectively (Fig. 3d). *L* can be calculated, assuming the suspended MoS_2_ surface has an arc shape. The height profile obtained from the AFM measurement was used to estimate the radius (435 nm) and the angle (32°) of the arc. The possible maximum strain in the MoS_2_ monolayer was estimated to be 1.2% from the height profile, assuming that the non-stretched MoS_2_ length (*L*_0_) was the same as the gap between the neighboring Au stripes (Fig. 3d). The widely used wet-transfer method was used to transfer the MoS_2_ monolayers, hence, water or other solvents could deform the MoS_2_ monolayer during the final drying process. The surface tension of the liquid could generate a force that drags the suspended region down to the substrate, resulting in uniaxial tensile strain^22^. Micro-photoluminescence (PL) and Raman measurements were carried out to study the optical properties of the MoS_2_ monolayers on the Au stripe arrays. The beam diameter in our PL and Raman systems was about 1 μm, which was two times larger than the period of our Au stripe array. Thus, clear difference in the PL and Raman spectra at the strained and non-strained regions could not be observed (see Fig. S1 of Supporting Information).

The AFM system was also used to measure the *V*_*S*_ of the sample in the amplitude-modulated KPFM mode^14,18,20^. The surface potential in dark (*V*_*S*,*D*_) was compared with that under light illumination (*V*_*S*,*L*_) using a light emitting diode module with 532 nm wavelength. The polarization of the laser light was adjusted to either transverse electric (TE) or transverse magnetic (TM) mode. The electric (magnetic) field of the TE- (TM-) mode light was parallel to the Au stripes. It has been known that the ambient gas adsorption can significantly vary the measured *V*_*S*_ data^11,12,14,18^. For example, adsorbed O_2_ molecules withdraw electrons from the MoS_2_ layer and dipoles formed by the O_2_^−^ ions decrease the *V*_*S*_^14,18^. To avoid such ambient-related artifacts, our KPFM measurements were done in a glove box purged with N_2_ gas.

**Figure 4:**
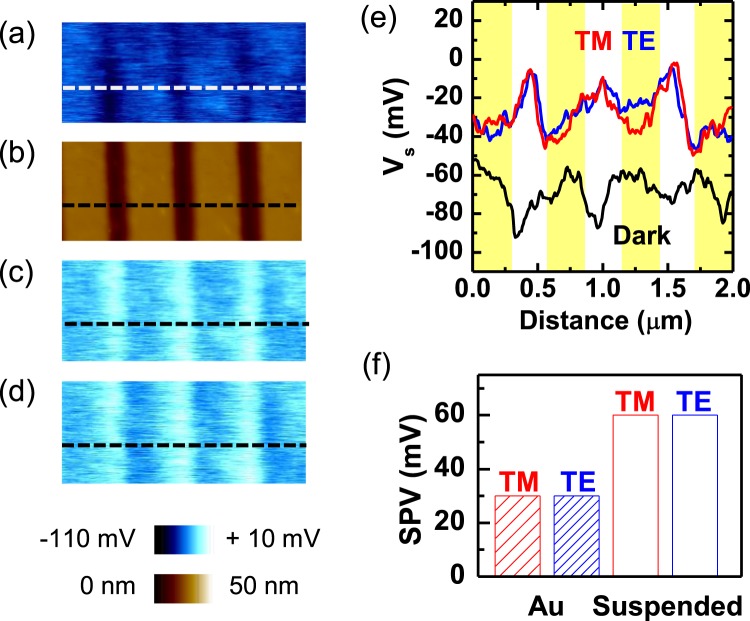
(**a**) A *V*_*S*,*D*_ map and (**b**) an AFM topography image of a MoS_2_ monolayer on the Au stripe array. *V*_*S*,*L*_ maps of the same region under illumination of (**c**) TM- and (**d**) TE-mode linearly polarized light. (**e**) Measured surface potential data as a function of distance along the dashed lines on the maps. The black, red, and blue lines represent the measured data in the dark, under TM-mode light, and under TE-mode light, respectively. The yellow bars indicate the region on the Au stripe. (**f**) The measured SPV data at the MoS_2_ surface on the Au stripe (indicated by ‘Au’) and the suspended MoS_2_ surface (indicated by ‘Suspended’) under illumination with TM (red) and TE (blue) mode light.

The *V*_*S*,*D*_ map of the MoS_2_ monolayer on an Au stripe array (Fig. 4a) shows spatial modulation with a period that was identical to that of the Au stripe array (Fig. 4b). The *V*_*S*,*D*_ measured on the MoS_2_ surface in contact with the Au stripe (*V*_*S*,*D*_-Au) was larger than that at the suspended MoS_2_ surface (*V*_*S*,*D*_-S), as shown in Fig. 4a. The measured *V*_*S*,*D*_-Au and *V*_*S*,*D*_-S were −50 mV and −90 mV, respectively (0 > *V*_*S*,*D*_-Au > *V*_*S*,*D*_-S). Interestingly, light illumination reverses the contrast in the *V*_*S*_ maps of our MoS_2_-Au nanostructure. Figure 4c and d are the *V*_*S*,*L*_ maps obtained from the same region in Fig. 4a under TM- and TE-mode light illumination (wavelength: 532 nm), respectively. Contrary to the *V*_*S*,*D*_ data, the *V*_*S*,*L*_ on the MoS_2_ surface on the Au stripe (*V*_*S*,*L*_-Au) was smaller than that on the suspended MoS_2_ surface (*V*_*S*,*L*_-S), in both kinds of linearly polarized light (0 > *V*_*S*,*L*_-S > *V*_*S*,*L*_-Au). The *V*_*S*_ profiles in Fig. 4e clearly show light-induced *V*_*S*_ modification in our sample. The difference between *V*_*S*,*D*_ and *V*_*S*,*L*_ is called as surface photovoltage (SPV), SPV = *V*_*S*,*L*_ − *V*_*S*,*D*_^18–20^. Figure 4f shows the measured SPV data of MoS_2_ on the Au stripe and the suspended MoS_2_ in TM- and TE-mode illumination. The SPV at the suspended region is larger and the polarization direction of the light does not cause a notable difference. If the excitation of the surface plasmon polariton occurs in our sample, the measured SPV and micro-reflectivity data should show a polarization dependence (see Fig. S2 of Supporting Information)^10^.

It has been well known that tensile strain can decrease the optical bandgap of monolayer MoS_2_^21–23^. The bandgap reduction increases the *V*_*S*,*D*_, as reported in the KPFM studies of Luo *et al*.^21^. They reported that the measured *V*_*S*_ difference in their 0.7%-strained MoS_2_ monolayer was up to 40 mV. The estimated strain in their sample was comparable to that of our sample. However, the *V*_*S*,*D*_-S was smaller than the *V*_*S*,*D*_-Au in our results (Fig. 4a). This shows that the strain-induced bandgap reduction alone cannot explain the *V*_*S*,*D*_ mapping results.

**Figure 5:**
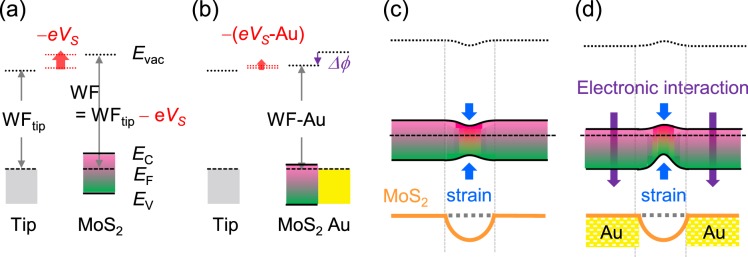
(**a**) Schematic band diagrams to explain the relation used in KPFM measurements, WF = WF_tip_ − *eV*_*S*_, where WF, WF_tip_, *e*, and *V*_S_ are the work function of a sample (MoS_2_), the work function of the tip, the electron charge, and the surface potential, respectively. *E*_vac_, *E*_C_, *E*_F_, and *E*_V_ are the vacuum level, conduction band minimum, Fermi level, and the valence band minimum of a sample, respectively. (**b**) The interfacial electric dipole energy, *Δϕ*, lowers the WF of the MoS_2_ region on the Au stripe (WF-S). It should be noted that *eV*_*S*_ = WF_tip_ − WF. The band diagrams in (**a**,**b**) indicate *V*_*S*_ < 0, consistent with our experimental results. Thus, reduction of WF increases *V*_*S*_ and decreases |*V*_*S*_| (*V*_*S*_ < 0), as illustrated in (**b**). Schematic band diagrams and illustration of the MoS_2_ monolayers on the Au stripe arrays in the dark, when including (**c**) only strain-induced bandgap reduction and (**d**) additional electronic interaction at the MoS_2_/Au interface.

Sohn *et al*. reported that the *V*_*S*_ of a MoS_2_ monolayer on an Au thin film was larger than that on a SiO_2_/Si substrate by 250 mV^14^. Strong electronic interaction at the MoS_2_/Au interface leads to electron wave function polarization and the formation of a large electric dipole^3,14^. Such interface dipole causes a potential drop (*Δϕ*) and increases *V*_*S*_^14^, as illustrated in Fig. 5a,b. In both Fig. 5a,b, it is assumed that the electronic affinity – separation between the vacuum level (*E*_*vac*_) and the conduction band maximum (*E*_*C*_) – does not change much. As a result, the Fermi level (*E*_*F*_) moves toward *E*_*C*_ in the MoS_2_ layer on an Au thin film. Such carrier concentration increase (i.e., doping) has been reported by both *V*_*S*_ and electrical transport measurements^3,14,18^.

Figure 5c shows the band diagram, when only strain-induced bandgap reduction is considered. The expected bandgap reduction is several tens of mV^21^, suggesting that the *V*_*S*,*D*_ at the strained region is larger than that at non-strained region. However, this is opposite to the experimental data (0 > *V*_*S*,*D*_-Au > *V*_*S*,*D*_-S). Figure 5d shows the band diagram including the electronic interaction as well as the local strain. The interface dipole energy is as large as a few hundreds of mV^14,18^, and hence the *E*_*C*_ in the MoS_2_ layer on an Au thin film is located below the *E*_*C*_ at the suspended MoS_2_ region. The band diagram in Fig. 5d agrees with our results: *V*_*S*,*D*_-Au is larger than *V*_*S*,*D*_-S (Fig. 4a,e).

Illumination with light can generate electron and hole pairs when the photon energy is larger than the bandgap energy of the sample. The electron and hole pairs can be separated by a potential gradient at the sample surface, if there is any. When the local sample surface is charged because of drift and diffusion of photo-generated charge carriers, a non-zero SPV can occur^14,19^. When only the strain effects are considered, both electrons and holes move toward the suspended MoS_2_ surface, where they both have a lower potential energy (Fig. 5c). In such a case, any change of the net charge density cannot be expected at the MoS_2_ surface. The band diagram in Fig. 5d suggests that electrons move toward the MoS_2_ surface on the Au stripe (i.e., the region with low potential energy) and holes move toward the suspended MoS_2_ surface under light illumination. Additionally, the electrons can be readily transferred from the MoS_2_ monolayer to the Au stripe, according to the band alignment at the MoS_2_/Au interface (Fig. 5b)^3,14^. Therefore, the resulting charge distribution leads to a higher positive charge density and a large SPV at the suspended MoS_2_ surface^19^. This scenario well explains all the experimental results. As discussed above, the dissociation and/or desorption of the gas adsorbates can influence the measured *V*s under light illumination^20^. The reversible recovery of the dark-state *V*_S_ showed that gas-adsorption/desorption-related *V*s variation could be avoided in our N_2_-ambient KPFM measurements (see Fig. S3 of Supporting Information).

## Conclusion

We fabricated MoS_2_-Au nanostructures, consisting of exfoliated MoS_2_ monolayers and Au nanostripe arrays with a period of 500 nm, and investigated their *V*_S_ maps using KPFM. The MoS_2_ monolayers were transferred on the 25-nm-thick Au stripes and the suspended MoS_2_ region between neighboring stripes underwent tensile-strain. The *V*_S_ at the suspended MoS_2_ region was smaller than at the region on the Au stripes in the dark. Such *V*_S_ difference could not be explained by the strain-induced bandgap reduction alone. The electronic interaction and resulting potential drop at the MoS_2_/Au interface should be taken into account to explain the *V*_S_ maps. Green light illumination reversed the contrast in the *V*_*S*_ maps of our MoS_2_-Au nanostructure: the SPV at the suspended MoS_2_ region (60 mV) was two times larger than that at the MoS_2_ region on the Au stripe (30 mV). Based on these observations, band diagrams were proposed and they successfully explained the experimental results. This work suggests that consideration of both local strain and electronic interactions at the MoS_2_/metal contact are crucial for realizing high-performance MoS_2_-metal nanostructure devices.

## Methods

### Surface potential measurements

We used Pt-coated Si cantilevers (ScanSens, NSG01/Pt) to measure the local surface potential (*V*_*S*_) in our KPFM system. *V*_*S*_ maps were obtained in dark (*V*_*S*,*D*_) and under light illumination (*V*_*S*,*L*_) using a light emitting diode module with 10 mW power and 532 nm wavelength (Laserlab, LDD532-10-5). The polarization of the laser light was adjusted with a linear polarizer (Thorlabs, LPVISC050-MP2) and a high-precision rotation mount (Thorlabs, MP2PRM05/M) to either TE or TM mode. We measured the topography and *V*_*S*,*D*_ at a specified area on the sample surface. For comparison, we measured the topography and *V*_*S*,*L*_ without moving the tip position. We identified the identical region from the images using relative coordinates from a clear topographic feature. The *V*_*S*,*D*_ after light exposure was compared with that before exposure to assure the recovery of the sample state its initial dark state.

### Optical simulations

Optical reflection spectra were obtained by finite-difference time-domain (FDTD) numerical calculations (Lumerical FDTD Solutions) and compared with the experimental results. A linearly polarized plane wave was used as a light source and the two kinds of polarization direction (TM- and TE-modes) were considered. In the calculations, the mesh size was 2 nm, which was much smaller than the thickness of the Au layer (20 nm). Cross-section monitor shows the electric field intensity map under illumination of normally incident light.

## Supplementary information

Supplementary information
