# Supplementary material for: Light-Induced Surface Potential Modification in MoS2 Monolayers on Au Nanostripe Arrays
Source: Sci Rep. 2019 Oct 8;9:14434. doi: 10.1038/s41598-019-50950-2 (PMC6783531; doi:10.1038/s41598-019-50950-2)
Supplement: Supplementary file 1 — Supplementary information [file 41598_2019_50950_MOESM1_ESM.pdf]

Supplementary Information

Light-Induced Surface Potential Modification in MoS_2 Monolayers on Au Nanostripe Arrays

Soyeong Kwon,[1] Min Hee Kwon,[1] Jungeun Song,[1] Eunah Kim,[1] Youngji Kim,[2] Bo Ra Kim,[1] Jerome K. Hyun,[2] Sang Wook Lee,[1] and Dong-Wook Kim[1,*]

[1] Department of Physics, Ewha Womans University, Seoul 03760, Korea

[2] Department of Chemistry and Nano Science, Ewha Womans University, Seoul 03760, Korea

* Correspondence and requests for materials should be addressed to D.W.K. (email: dwkim@ewha.ac.kr)

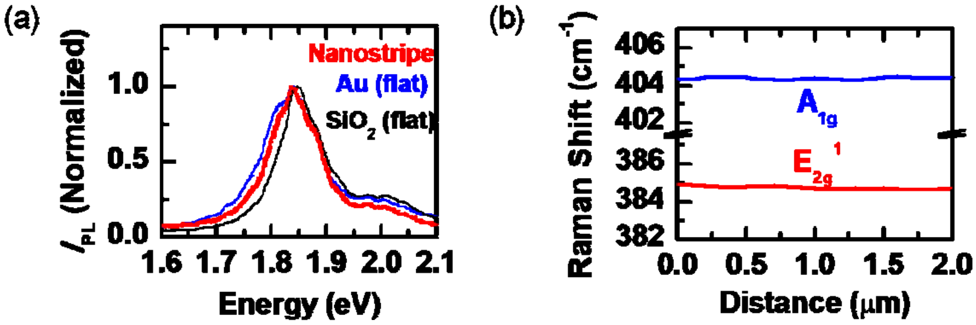

Figure S1. (a) Normalized micro-photoluminescence (PL) intensity spectra of three kinds of samples: MoS_2 monolayers on the Au nanostripe array (red), a flat Au thin film (blue), and a flat SiO_2/Si substrate (black). (b) The E^1_{2g} and A_{1g} mode peak locations of the MoS_2 monolayer on the Au stripe array as a function of distance of the laser beam spot during the micro-Raman measurements. The laser beam was scanned across the stripe array (data acquisition step: 0.1 μm). A 532-nm-wavelength laser source with the beam diameter of ~ 1 μm was used for both of the PL and Raman measurements.

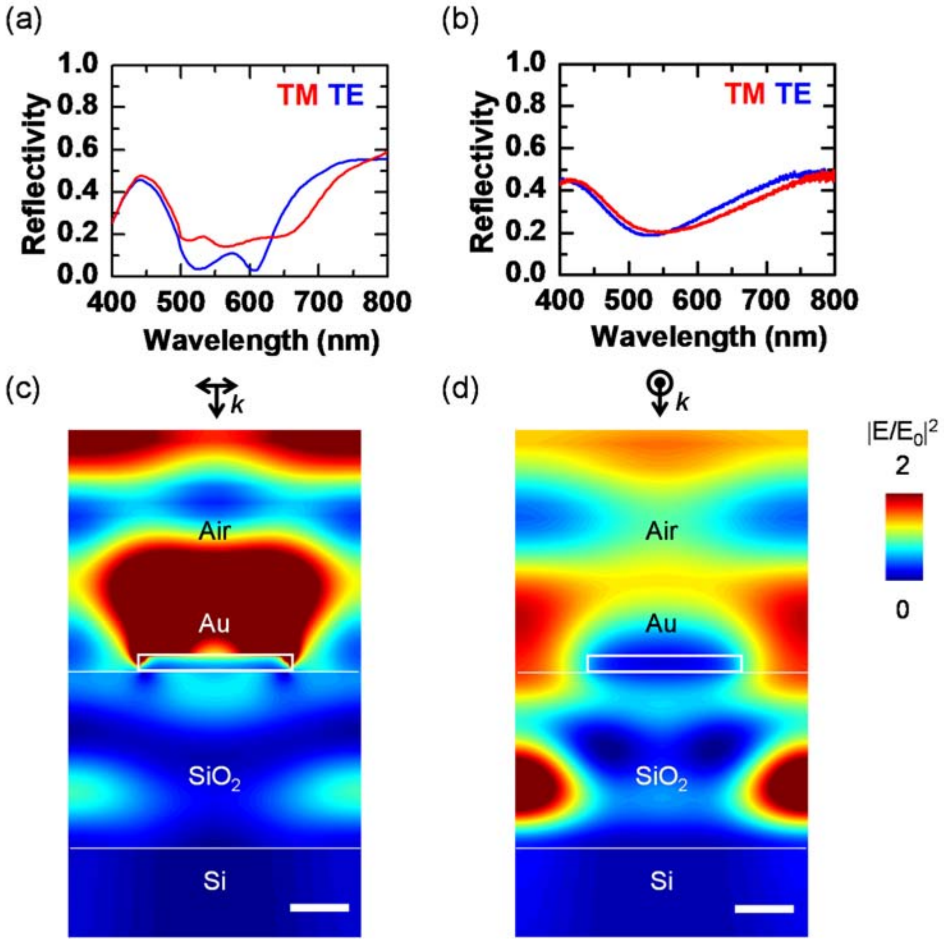

Figure S2. Reflectivity spectra of the Au stripe array, obtained by (a) finite-difference time-domain (FDTD) calculation and (b) measurement, for normal incident TM- and TE-mode light illumination. A home-built microscope coupled to a spectrometer was used to measure the reflectivity. The beam spot size was as small as 2-3 μm. The cross-sectional electric field intensity distribution maps from the FDTD simulations under (c) TM- and (d) TE-mode light at a wavelength of 532 nm (scale bar: 100 nm). Both calculations and measurements did not show any clear distinction between the TM- and TE-mode reflectivity spectra. No clear polarization dependence suggests that surface plasmon polariton excitation is not expected in our stripe array sample.

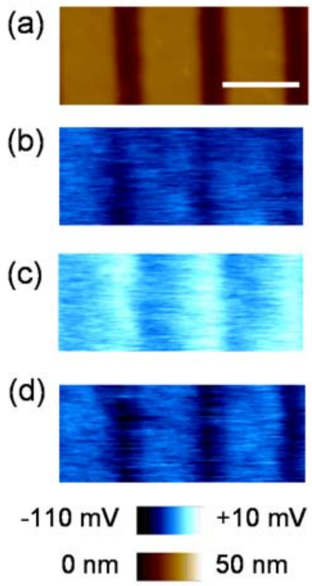

Figure S3. (a) An AFM topography image of a MoS_2 flake on an Au stripe array, (b) a $V_{S,D}$ map, (c) a $V_{S,L}$ map under illumination of TE-mode light, and (d) a $V_{S,D}$ map after the light exposure (scale bar: 0.5 μm). The $V_{S,D}$ maps in (b) and (d) were obtained before and after the $V_{S,L}$ mapping measurement in (c), respectively. No notable difference can be seen in the $V_{S,D}$ maps before and after the light illumination. If light-induced gas desorption and resulting surface potential change occur, such recovery of the $V_{S,D}$ cannot be observed.
